# The Function of Astrocyte Mediated Extracellular Vesicles in Central Nervous System Diseases

**DOI:** 10.3389/fcell.2020.568889

**Published:** 2020-10-15

**Authors:** Tahereh Gharbi, Zhijun Zhang, Guo-Yuan Yang

**Affiliations:** Shanghai Jiao Tong University Affiliated Sixth People’s Hospital, School of Biomedical Engineering, Shanghai Jiao Tong University, Shanghai, China

**Keywords:** astrocyte, brain, extracellular vesicle, exosome, stem cell

## Abstract

Astrocyte activation plays an important role during disease-induced inflammatory response in the brain. Exosomes in the brain could be released from bone marrow (BM)-derived stem cells, neuro stem cells (NSC), mesenchymal stem cells (MSC), etc. We summarized that exosomes release and transport signaling to the target cells, and then produce function. Furthermore, we discussed the pathological interactions between astrocytes and other brain cells, which are related to brain diseases such as stroke, Alzheimer’s disease (AD), Parkinson’s disease (PD), amyotrophic lateral sclerosis (ALS) disease, multiple sclerosis (MS), psychiatric, traumatic brain injury (TBI), etc. We provide up-to-date, comprehensive and valuable information on the involvement of exosomes in brain diseases, which is beneficial for basic researchers and clinical physicians.

## Introduction

Astrocytes are the most abundant glial cells in the human brain. Astrocytes play key roles in the brain such as maintenance and formation of the blood-brain barrier (BBB) (Abbott et al., 2006), providing metabolic support to the nervous tissue (Benarroch, 2005; Allaman et al., 2011), regulation of regional blood flow (Attwell et al., 2010; Bazargani and Attwell, 2016), regulation of synaptic circuits (Dallerac et al., 2018), neurotransmitter recycling (Hamilton and Attwell, 2010) as well as repairing and scarring process of the brain and spinal cord following various injuries (Anderson et al., 2014; Adams and Gallo, 2018).

The diversity and essence of the functions of astrocytes make them predominant among other cells in the central nervous system. Previous studies have demonstrated various inflammatory, anti-inflammatory, neuroprotective and disease-causing effects of astrocytes in different diseases (Gupta et al., 2012; Sofroniew, 2015; Habib et al., 2020). Hundreds of researches have been conducted to increase our understanding of how astrocyte exert their effects. However, the ways astrocytes influence other cells’ functions remain largely unknown.

During the past decade, scientists found that extracellular vesicles (EVs) play important roles in both short and long-distance communications between cells within and out of the brain (Simons and Raposo, 2009; Zhuang et al., 2011; Chen et al., 2016; Paolicelli et al., 2019; Kur et al., 2020). Astrocyte-derived EVs are enriched with various biological molecules including genes, microRNAs (miRNA), and proteins. Interestingly, astrocytes-derived EVs that are secreted in normal conditions are known to be enriched with neuroprotective and neurotrophic elements. On the contrary, EVs released by astrocytes under abnormal conditions such as oxidative stress, nutrient deficiency, inflammation have been witnessed to exert neuroprotective effect and promote neurite regeneration and outgrowth (Taylor et al., 2007; Wang et al., 2011; Moidunny et al., 2012; Gosselin et al., 2013). In response to neuronal inflammation, astrocytes modify their EV component to suppress the inflammation by regulating the excitability of neurons (Chaudhuri et al., 2018). Astrocytes also modify their EV contaminations in response to extracellular stimuli. For example, upon normal extracellular stimuli, astrocyte-derived EVs contain proteins that promote neurite outgrowth, synaptic transmission, and neuronal survival. Whereas, when exposed to an inflammatory stimulus, Interleukin 1beta (IL-1β), astrocyte shed EVs that regulate peripheral immune response (Dickens et al., 2017; Chaudhuri et al., 2018; Datta Chaudhuri et al., 2020). Not only do astrocytes respond to stimulus and conditions by secreting EVs with selected cargos, but they are also influenced by EVs secreted from other cells. Researches have illustrated that when a specific neuronal exosomal miRNA is internalized into astrocyte, it can change the astrocytic function from MSCs to astrocytes and neurons regulate gene expression of astrocytes, thus improving functional recovery and neurite regeneration after stroke (Xin et al., 2013; Men et al., 2019). Unfortunately, astrocytes could also be influenced by cancer cells which can lead to devastating effects. Glioblastoma EVs alter the astrocytes to increase tumor growth and may drive astrocytes to a tumorigenic phenotype (Oushy et al., 2018). Experiments also demonstrated that astrocyte may evoke changes in glial cells’ responses that could be elicited by EVs only (Willis et al., 2020b). Overall, various EVs seem to play a key role in cell to cell communication in both healthy and disease conditions. EVs are undoubtedly one of the most important factors if not only in facilitating communication between astrocytes and other cells. In this review, we summarize the functions of astrocyte-derived EVs and other brain cell-derived EVs.

Extracellular vesicles, lipid-bound vesicles that are crucial in intracellular communication, are secreted into extracellular space. EVs exist in variety of size, content, origin and targets. EVs are mostly classified.

**FIGURE 1:**
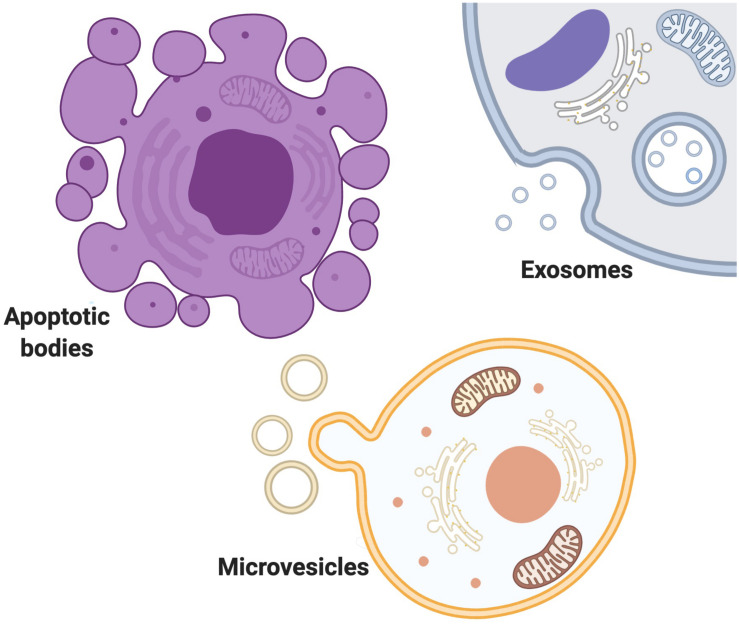
Extracellular vesicles exist in variety of size, content, origin and targets. EVs are mostly classified Into three main subtypes of MVs, apoptotic bodies and exosomes. MVs are secreted by outward budding and splitting of plasma membrane. Apoptotic bodies are produced by blebbing of apoptotic cells. Exosomes are formed as infusion of multivascular body with plasma membrane.

Into three main subtypes of microvesicles (MV), apoptotic bodies and exosomes (Borges et al., 2013; Yanez-Mo et al., 2015; Zaborowski et al., 2015) (Figure 1). EVs are loaded with different proteins, lipids, and nucleic acids (Sedgwick and D’Souza-Schorey, 2018). MVs are known as shedding vesicles, ectosomes, microparticles are more heterogenous and bigger in size (100 nm^–1^μm in diameter) (Raposo and Stoorvogel, 2013; Pollet et al., 2018). MVs are secreted by outward budding and splitting of plasma membrane. Apoptotic bodies are 1–5μm in diameter and are produced by blebbing of apoptotic cells (Raposo and Stoorvogel, 2013; Sedgwick and D’Souza-Schorey, 2018). Exosome was first discovered as a vesicle in the immature red blood cells in 1983 (Harding and Stahl, 1983; Pan and Johnstone, 1983), and further named “exosome” by Johnstone et al. (1987). exosomes are smaller than a bacterium and exist in various sizes from 30 to 100 nm in diameter and are formed as infusion of multivascular body with plasma membrane (Borges et al., 2013; Raposo and Stoorvogel, 2013). Exosomes are not only a way of waste disposal, but they are also released to induce an effect on their targets by making them active. Cells could utilize exosomes to send genetic signals to other cells (Nagarajah, 2016). Exosomes and other EVs mediate intracellular communication between various cells by carrying a diverse quantity of bioactive molecules (Camussi et al., 2010; Miyabe et al., 2019). The importance of the exosome-mediated intercellular communication compared with the traditional modulation factors like such as hormones, cytokines, electrical and chemical signals and etc is that communication through exosomes within the cells is highly reliant on the delivery of genetic biomaterials which can greatly and impact on cells behavior and phenotype changes (Ratajczak et al., 2006; Valadi et al., 2007; Skog et al., 2008; Mittelbrunn et al., 2011; Montecalvo et al., 2012; Zhang Y. et al., 2019), whereas Traditional modulation factors act on receptors or ligands to affect recipient cell functions (Premack and Schall, 1996; Thelen, 2001; Tran and Miller, 2003; Brooks and Waters, 2010; Camussi et al., 2010; Levin and Hammes, 2016; Miyabe et al., 2019; Van der Auwera et al., 2020). Communication through exosomes is more complex than through secretion of chemokines and cytokines because of exosome’s proper packaging of biomaterials and the study of exosomes may help in identifying disease root cells in pathophysiology (Rooj et al., 2016). Exosomes could be released into body fluids and travel to the targeting tissue. After being taken up by specific cells, they could subsequently change the function and status of the recipient cells (Salido-Guadarrama et al., 2014). Based on the genetic materials they carry, they develop a certain ability to interact with different cells (Zhang Y. et al., 2019). Exosomes play a significant role in many biological processes in both healthy and abnormal cells (Kalluri, 2016; Samanta et al., 2018; Saeedi et al., 2019; Holtzman and Lee, 2020; Ni et al., 2020; Zhou et al., 2020). The functions of exosome’s and other EVs are important in both diagnosis and therapeutics clinically due to their diverse effects on various diseases and conditions (Xu et al., 2018). In terms of therapeutic functions, Evs’ potential involvement in the treatments is promising as they could migrate toward the target cells precisely with lower adverse effects and toxicity (Schorey and Bhatnagar, 2008; Lin et al., 2019; Thery et al., 2018; Qiu et al., 2019). Many reasons make EVs predominant among other therapeutic factors in disease therapy. EVs can pass the BBB, so it is useful in the treatment of central nervous system (CNS) disease (Zhuang et al., 2011; Liu et al., 2015; Khongkow et al., 2019). EVs can be loaded with biomaterials and targeted at certain types of disease-causing cells such as tumor cells (Hao et al., 2007; Luan et al., 2017; Yang and Li, 2018; Walker et al., 2019; O’Brien et al., 2020). EVs derived from stem cells can also be useful for neuro-regeneration after CNS injuries (Luarte et al., 2016; Holm et al., 2018; Vogel et al., 2018; Branscome et al., 2020). Identification and inhibition of disease-causing EVs can also be a potential therapeutic strategy in the treatment of various diseases (EL Andaloussi et al., 2013). Scientists seek to turn the exosomes into a promising factor in the diagnosis of brain cancer to make it possible to obtain liquid samples instead of performing brain biopsy in the early and curable stages of brain cancer diagnosis (Manterola et al., 2014; Santiago-Dieppa et al., 2014). Recently, scientists also established a method by using a smartphone to detect enzymatic exosomes after brain injury in concussion diagnosis (Ko et al., 2016).

Exosomes could simply present the cell genetic information and from that, exosomes could help to distinguish the abnormal cells from normal and healthy cells (Isola and Chen, 2017). The significance of exosomes in the cell to cell communication and transportation of unique genetic materials could be considered as biomarkers in the diagnosis and therapy of various diseases. Cancer studies showed that exosomes can be considered as potential biomarkers in diagnosis and prognosis of cancer (Wong and Chen, 2019). Besides, it was also revealed that DNM3, p65, and p53 followed a dysregulated-patterns in the brain and blood exosomes in both original and recurrent stages of glioblastoma multiforme. DNM3, P65, and P53 enriched exosomes can be a potential clinical diagnostic marker for glioblastoma multiforme (Yang J.K. et al., 2017). Altogether, studies suggest that exosomes have high sensitivity and specificity in the assessment of cancer and they have the potential to be considered as biomarkers in the diagnosis and prognosis of cancers and other diseases (H Rashed et al., 2017; Abak et al., 2018).

There are also obstacles in the study of EVs *in vivo*. EVs are recently discovered particles in many diseases. How to study specific EV derived from a certain type of cell is still unclear and, how to prove the importance of EVs in the cell to cell communication is still an obstacle. Nevertheless, the role of astrocyte-derived EVs in both health and disease conditions have been summarized in our review.

## Importance of Astrocyte-Derived EVs

Astrocytes are known to be involved in brain homeostasis regulation (Hubbard et al., 2016; Verkhratsky and Nedergaard, 2018; Jha et al., 2019). However, the underlying mechanisms of how astrocytes communicate with other cells in the brain microenvironment to regulate brain homeostasis is still unclear. The brain is capable to communicate with the rest of the body via transporting EVs to communicate healthy and disease states of the body (Gomez-Molina et al., 2019). Study of exosomes could increase our knowledge of astrocytes, their homeostasis, and metabolic regulating mechanisms since they are capable of transferring biological molecules from one cell to another (Fruhbeis et al., 2013; Silverman et al., 2019; Upadhya et al., 2020; You et al., 2020). Interestingly, miRNAs contained in the exosomes secreted by mouse astrocytes were different from the miRNAs that originally existed in mouse astrocytes. These findings suggest a mechanism that astrocytes has to select a specific miRNA for excretion via exosomes (Jovicic and Gitler, 2017). Astrocytes may also secrete EVs to affect surrounding cells in a positive or negative way. Astrocyte shedding vesicles containing miR-34a increased the sensitivity of dopaminergic neurons to neurotoxins (Mao et al., 2015). Under abnormal conditions, astrocytes are triggered to releases immune-related or anti-inflammatory related vesicles in response to stress-related stimuli. In contrast, in a normal situation, astrocyte-derived EVs promote neurite outgrowth and survival (Datta Chaudhuri et al., 2020).

Another research demonstrated that astrocytes may support oligodendrocyte differentiation by EVs, however, this support disappears with age (Willis et al., 2020a). Identification of specific stimulus that astrocytes respond to by secreting EVs may increase our understanding of CNS diseases or aged brain. Astrocyte-derived EVs contain protein biomarkers that are identified to be involved in stress-induced diseases. Besides, astrocyte-derived exosomes could maintain cell-specific markers, which allow the identification of the origin of parental cells, disease initiation and progression. Released exosomes from the cells can be isolated from the tissue and then be examined as an assessment for the identification of cellular origins of extracted exosomes from body fluids for diagnostic purposes such as blood, saliva, and urine in finding the disease-related type of cells (Venturini et al., 2019). Astrocyte-derived EVs can also be useful in drug targeted therapies as a drug or drug carrier because they can pass the BBB and be taken up by brain cells. Astrocyte-derived EVs containing Cox2 small interfering RNA was capable of restoring microglial phagocytic activity after being up-taken by microglial cells in morphine-mediate neurodegenerative mice model (Hu et al., 2018). In general, obtaining a better understanding of EVs secreted from astrocytes may improve CNS disease diagnosis as well as drug targeting therapies.

## The Relationship Between Astrocyte-Derived EVs and Microenvironment

**FIGURE 2:**
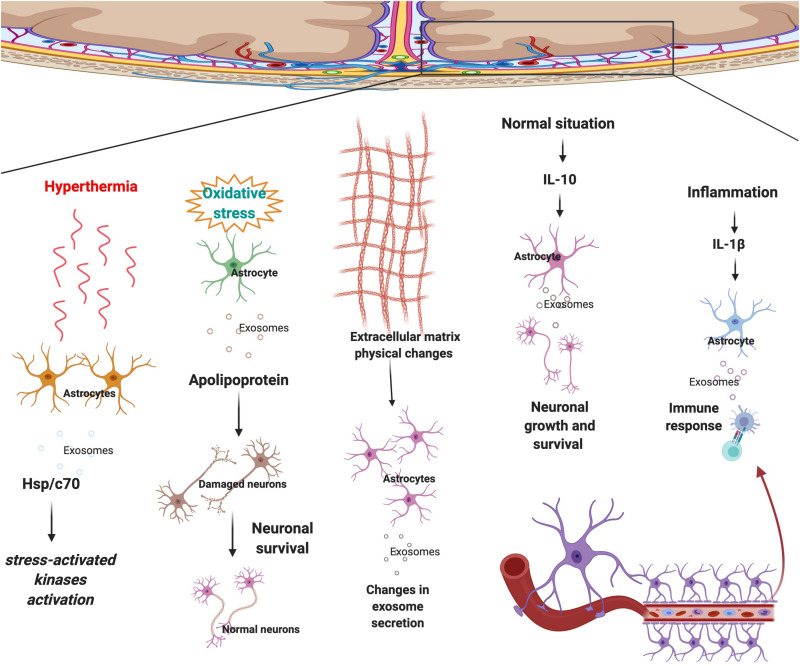
Astrocytes respond to surrounding signals and physical changes in the microenvironment by sending selected exosomes to maintain brain homeostasis. In response to hyperthermia astrocytes secrete exosomes that contain Hsp/c70 and further activate stress-activated kinases to lower the stress. Upon oxidative stress, EVs secreted by astrocytes transport apolipoprotein, a classical neuroprotective protein, to neurons and mediate neuronal survival. Physical changes to ECM morphology also affect astrocyte-derived EV production and possibly cell to cell communication. Following secretion of IL-10 in the normal condition, an anti-inflammatory signal, astrocyte-derived EVs contain proteins that lead to neuronal survival. Whereas, in regards to IL-1β, an inflammatory signal, astrocyte-derived EVs contain proteins that mediate immune response.

Similar to the other cells, astrocytes are surrounded by various structures and cells such as neurons, oligodendrocytes, microglia, micro-vessels, extracellular matrix (ECM), etc and they interact with each other regularly. Astrocyte-derived exosomes are one of the most significant ways of communication between astrocytes and surrounding cells. Physical or chemical microenvironmental changes affect astrocytes’ growth and behavior. Changes in astrocytes alter the type of signals they release, therefore altering the microenvironment in a positive or negative manner (Paolicelli et al., 2019; Schiera et al., 2019; You et al., 2020). A study found a novel brain penetrant inhibitor that inhibited the release of vesicles in astrocytes (Rojas et al., 2019). Recently, microenvironmental factors such as ECM, immune cells and blood were frequently studied. Microenvironmental signals play various roles in different disease initiation and progression. Changes in microenvironments could affect the communication of astrocytes with other cells. Previous studies also revealed that astrocyte secrete EVs in response to the stimuli/signals coming from the environment. The mobility of astrocyte-derived vesicles in rats increased in response to the release of ALS-IgG (Stenovec et al., 2011). Which illustrates that astrocytes derived-EVs and microenvironment influence each other regularly. For example, in response to interleukin 10 (IL-10), astrocyte-derived EVs contain proteins that lead to neuronal survival (Figure 2). Whereas, in response to IL-1β, astrocyte-derived EVs contain proteins that mediate immune response (Datta Chaudhuri et al., 2020) (Figure 2).

Central nervous system disease progression and initiation are not necessarily the result of genetic mutations, physical changes in the microenvironment could also cause the disease by altering astrocyte-derived EVs content and secretion. Recent research aimed to investigate the impact of scaffold morphology on the astrocyte growth and their colonization ability by mimicking ECM. As a result, it was found that physical changes in the microenvironment could affect astrocyte-derived EV production and possibly cell to cell communication (Carfi Pavia et al., 2019) (Figure 2). From this research, it is evident that astrocytes change the exosomes’ selection strategy in response to the physical changes in the micro environment. Another study demonstrated that astrocytes that are undergoing hyperthermia increased the releasing amount of heat shock protein/c70 (Hsp/c70) via exosomes into the extracellular environment following stress (Taylor et al., 2007). The study also showed that all the extracellular Hsp/c70 secreted by astrocytes were present in exosomes, implying the importance of exosome release in astrocytic function under stress conditions (Taylor et al., 2007) (Figure 2).

### Stem Cell Derived Exosome Is Neuro Protective via Astrocytes

Stem cell therapies have been a famous treatment option for various CNS diseases (Lee et al., 2017; Wei et al., 2017; Sonntag et al., 2018; Sugaya and Vaidya, 2018; Zhang et al., 2018).

Recently, therapeutic abilities of stem cells in healing various CNS disease was referred to their ability of generating EVs (Yang Y. et al., 2017; Bang and Kim, 2019; Ni et al., 2019; Riazifar et al., 2019; Zhang Z.G. et al., 2019; Zhu et al., 2019; Ghosh et al., 2020). Some evidence show that stem cells may exert their effect via communication with astrocytes.

Neuro stem cells (NSCs) are known to be effective in tissue regeneration and repair in various CNS diseases (Marsh and Blurton-Jones, 2017; Ludwig et al., 2018). NSC applied its anti-inflammatory and neurotrophic effects by secreting EVs (Schindowski et al., 2008; Webb et al., 2018; Rong et al., 2019). Mouse NSC-derived exosomes have the ability to protect astrocytes by preserving their functions post-ischemic stroke (Sun et al., 2019).

**FIGURE 3:**
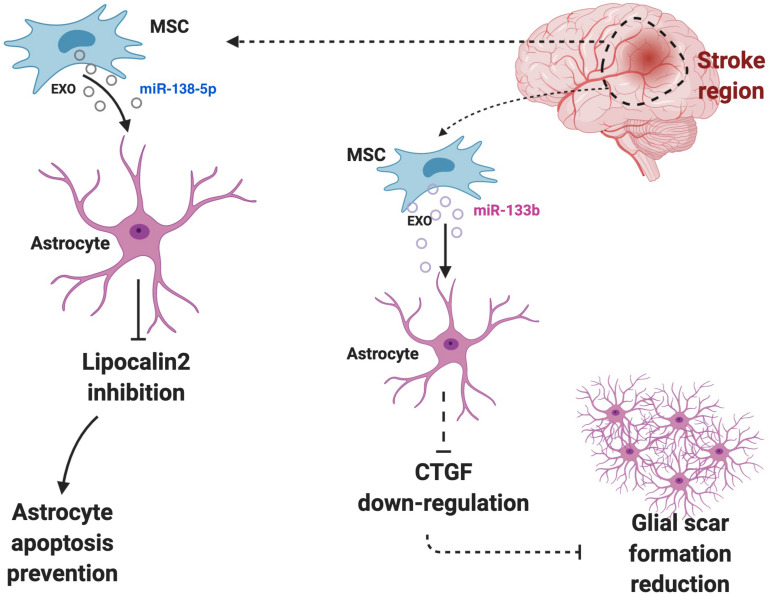
Astrocytes and surrounding cells in the microenvironment regularly communicate and interact with each other via exosomes. Cells may use astrocytes to exert their effects. For instance, BM-derived stem cells miR-138-5p-overexpressing exosomes prevent astrocytes from apoptosis after ischemic stroke by targeting lipocalin 2 or high level of miR-133b in astrocytes can down-regulate the expression of CTGF, which cause glial scar reduction and further increase the recovery post-ischemic stroke.

Evidence demonstrated that BM-derived stem cell exosomes could affect astrocytes’ functions. BM-derived stem cells are famous for being effective in the treatment of various diseases, especially extraosseous diseases, and are known to exert their effect by releasing exosomes (Lyu et al., 2020). It was also demonstrated that EVs secreted by BM-derived stem cells could transfer selected miRNAs (Collino et al., 2010; Baglio et al., 2015). BM-derived mesenchymal stem cells (MSCs) could also be an effective therapeutic for the treatment of diabetes-induced cognitive impairment through shuttling exosomes to astrocytes and neurons that can increase the recovery of astrocytes and boost neuronal functions (Nakano et al., 2016). BM-derived stem cells miR-138-5p-overexpressing exosomes could prevent astrocytes from apoptosis after ischemic stroke by targeting lipocalin 2 (Deng et al., 2019) (Figure 3). The miR-146a overexpression in BM-derived MSC exosomes can be triggered by an enriched environment, and it could inhibit inflammation in damaged astrocytes and prevent diabetes-induced cognitive impairment (Kubota et al., 2018).

Multipotent MSCs regulate neurite outgrowth and exert other effects by releasing EVs containing key molecules (Xin et al., 2012).

MSC-derived exosomes transfer miR-133b to astrocytes and MSCs derived exosomes elevate its miR-133b level after ischemic stroke. The high level of miR-133b in astrocytes then can down-regulate the expression of connective tissue growth factor (CTGF), which could cause glial scar reduction and further increase the recovery post-ischemic stroke (Xin et al., 2013) (Figure 3). Besides, it could improve the neuronal recovery after ischemic stroke by down-regulation of rab9 Effector Protein With Kelch Motifs (RABEPK) expression in astrocytes. MSC-derived exosomes could be used in the treatment of brain disease though affecting astrocytes functions by exerting an anti-inflammatory effect. A study identified that the nuclear factor (erythroid-derived 2)-like 2 and nuclear factor-κB (Nrf2-NF-κB) signaling pathway involved in an astrocytic activation and MSC-exosomes lowered the inflammation that causes astrocytic alteration to neurotoxic astrocytes (Xian et al., 2019). The affect of MSC-derived exosomes on neurotoxic A1 astrocytes induced by an inflammatory response in post-spinal cord injured patients. Their result demonstrated that MSC-derived exosomes could exhibit an anti-inflammatory effect on spinal injury and further decrease A1 astrocytes (Wang et al., 2018).

Finding the specific exosomes involved between astrocytes and stem cells toward the specific glial cells is a promising strategy in the brain healing process in various brain diseases and help in understanding the brain development and differentiation as well as *in vitro* brain development. Study of astrocyte-like cell-derived EVs and their effect on inducing the differentiation of neural stem progenitor cells toward glial cells *in vitro* experiment may confirm the importance of exosomes in cellular communication in neuron activation during development (Stronati et al., 2019).

## Astrocytes, EVs and CNS Diseases

Astrocyte-derived EVs are highly diverse in size, composition, and function. This may be evidence of why astrocytes could have both beneficial and pathological effects in various CNS diseases. Recent studies on astrocyte-derived exosomes have demonstrated the astrocytic roles in homeostasis and the possible relationship of homeostasis disruption with neurological diseases. Astrocyte-released exosomes may contain proteins with neurotrophic properties such as co-chaperone STI1. Astrocyte-derived exosomes that contain STI1 could be involved in brain injury, cancer, and neurodegenerative disorder (Hajj et al., 2013). Astrocytes may also utilize exosomes to play their role in regulating homeostasis in CNS, disruption of astrocytic balance in expressing amino acid transporters and protein kinases could relate to neurological diseases (Gosselin et al., 2013). The involvement of astrocytes and EVs in CNS diseases are summarized below. The highlighted molecules enriched EVs that are involved in disease progression and inhibition are summarized in Table 1.

**TABLE 1:** Extracellular vesicles enriched molecules involved in CNS diseases.

Disease	Mechanism	Involved molecule in EV	References
Brain injury, cancer, neurodegenerative disorders	Astrocyte release exosomes have neurotropic effects	STI1	Hajj et al., 2013
Inflammation	Astrocyte-derived EVs lower neurite development and maturation during inflammation	IL-1β	You et al., 2020
Stroke	MSCs transfer EVs to astrocytes and neurons and consequently lead to neuronal remodeling Astrocyte-derived exosome protects neurons after cerebral ischemia	miR-133b miR-34c	[Bibr B165][Bibr B162]
Oxidative stress and neurodegenerative diseases	EVs secreted from astrocytes to neurons protect neurons from oxidative stress	Apolipoprotein D	Pascua-Maestro et al., 2018
AD	Astrocyte release exosomes is triggered by Amyloid peptide cause apoptosis in astrocytes Amyloid-β exposed astrocytes overproduced exosomes	PAR-4/ceramide Phosphor-Tau protein	[Bibr B153][Bibr B26]
ALS	Brain astrocytes release EVs that may be involved in ALS disease formation Astrocyte-derived exosomes level of IL-6 elevated with the disease progression	SOD1 IL-6	Basso et al., 2013; [Bibr B128][Bibr B23]
PD	Astrocyte-derived exosomes prevents MPP^(+)^-induced apoptotic cell death through down-regulation of MKK4 pathway AND Protect neurons	miR-200a-3p	Shakespear et al., 2020
Brain cancer metastasis	Astrocyte-derived EVs increase brain cancer metastasis through the loss of PTEN in tumor cells and CCL2 chemokine secretion	miR-19a	Zhang et al., 2015
Glioma	Tumor-associated astrocytes derived EVs promote anti-tumor resistance in glioma cells Glioma cell secrete exosomes that cause phenotype changes in astrocytes and further promote glioma invasion.	MGMT mRNA Long non-coding RNA (lncRNA) activated by TGF-β	[Bibr B174][Bibr B13]
SCI	Astrocyte may release exosome containing proNGF in triggering neuronal apoptosis and involve in worsening SCI	proNGF	Cheng Y.Y. et al., 2019
TBI	Astrocyte secrete EVs could protect and repair damaged neurons by attenuating the CX43 phosphorylation and protecting the mitochondria, decreasing cell death rate and increasing neuronal recovery	GJA-20k	Chen W. et al., 2019

### Stroke and Ischemia

Astrocytes could exhibit both protective and negative effects on neurons after stroke. In the acute phase, reactive astrocytes play a neuroprotective role by decreasing the lesion extension, anti-excitotoxicity effects, and secretion of neurotrophins, whereas they may have a negative or positive effect in the chronic phase (Liu and Chopp, 2016; Pekny et al., 2019). Researches confirmed the positive effect of astrocyte-derived exosomes on neurons before and after ischemic stroke. Astrocyte-derived exosomes transfer miR-92b-3p to neurons before ischemia to protect neurons (Xu et al., 2019).

Astrocyte-derived exosomes were capable of suppressing autophagy and improving damaged neurons after ischemic stroke (Pei et al., 2019). Astrocyte-derived exosome containing prostaglandin _*D*2_ synthase lead to axonal outgrowth and increase the recovery after stroke (Hira et al., 2018). Astrocyte-derived exosome containing miR-34c protects neurons after cerebral ischemia (Wu et al., 2020). Astrocyte-derived exosome containing miR-361 protects nerve damage after stroke by mediating the AMPK/mTOR signaling pathway (Bu et al., 2020).

An *in vitro* study showed that oxygen-glucose depletion (OGD) in astrocytes-derived exosomes enriched miR-133b mediate neuron outgrowth and elongation after stroke (Xin et al., 2013). Astrocyte-derived exosomes also shuttle miR-190b to prevent OGD-induced autophagy and to inhibit neuronal apoptosis (Pei et al., 2020).

Under hypoxic and ischemic conditions, astrocytes release exosomes containing prion proteins that improve neuronal survival after being uptaken by neurons. Astrocyte-derived exosomes increase neuronal survival under oxidative and ischemic stress (Guitart et al., 2016). Cortical neuron-derived exosomes containing miR-181c-3p have been shown to inhibit neuroinflammation in an ischemic brain injury rat model by downregulating chemokine (C-X-C motif) ligand 1 (CXCL1) in Astrocytes (Song et al., 2019).

Extracellular vesicles secreted by astrocytes transport apolipoprotein, a classical neuroprotective protein, to neurons and mediate neuronal survival upon oxidative stress (Pascua-Maestro et al., 2018) (Figure 2).

### Alzheimer’s Disease

Alzheimer’s disease is caused by amyloid plaques containing amyloid-βpeptidases and neurofibrillary tangles containing tau protein. Neuronal death and cerebral amyloid angiopathy is commonly witnessed in AD patients. Investigations of fluid biomarkers in astrocytes and neuronal cells have proved that degenerative and inflammatory factors in early and late stages of AD are different and identifying the level of degenerative and inflammatory factors in both healthy and various stages of neurodegenerative diseases may help us to evaluate our understandings toward disease-causing factors such as inflammation, cellular plasticity, neuronal injury and astrocytopathy (Elahi et al., 2019). Neuronal-derived exosomes enhance the formation of Amyloid-β fibril and the exosomes containing Amyloid–β are taken into microglial for degradation (Yuyama et al., 2012).

Amyloid peptide trigger secretion of proapoptotic exosomes and may lead to glial apoptosis in AD (Wang et al., 2012).

A recent study demonstrated that Amyloid–β exposed astrocytes overproduced the phosphor-Tau protein within exosomes, which was a biological marker of AD (Chiarini et al., 2017). Another research revealed that amyloid–β and tau released into serum were most likely from astrocytes-derived exosomes in the brain according to animal experiments (Rosas-Hernandez et al., 2019). The disease-causing mechanisms of astrocytes are still unknown. Astrocytes are transformed into A1-type astrocytes which results in neuronal cell death and damages in AD. Neurotrophic factors levels released by astrocyte exosomes were lower at the early stage of AD with no further depletion of neurotropic levels by the progression of the disease in the later stages (Goetzl et al., 2019). The number of complement proteins in plasma astrocyte-derived exosomes are usually irregular in AD and are not the same in mild cognitive impairment (Winston et al., 2019). A study explored the inflammatory roles of astrocyte-derived exosomes by quantification of their complement proteins and they revealed that the complement proteins were the product of dysregulated unknown mechanisms, which damaged neurons in the late stage of AD (Goetzl et al., 2018). Because of the diversity of exosomes and the biological molecules they carry as a signal to other cells, exosomes may also be involved in disease-preventing mechanisms. Quantification of astrocyte-derived exosome proteins in AD and frontotemporal dementia suggests that exosome is a promising target for testing inhibitory drugs in proteinopathic dementia diseases (Goetzl et al., 2016) and indicates that astrocyte-derived exosomes may participate in the pathophysiology of AD (Khongkow et al., 2019). The robust effect of astrocyte-derived EVs in AD patients can also show the usefulness of astrocyte-derived EVs in brain targeting therapies (Nogueras-Ortiz et al., 2020).

### Amyotrophic Lateral Sclerosis Disease

Amyotrophic lateral sclerosis (ALS) is the most common motor neuron disease. It is an incurable disease because the pathways that lead to selective motor neuron damage are yet unknown. Astrocytes play a significant role in ALS (Basso et al., 2013). The brain astrocyte-derived EVs from ALS animals contain ALS causing proteins such as superoxide dismutase1 (SOD1), which are only found in patients with early-stage ALS (Silverman et al., 2019). Another study indicated that astrocyte-derived exosomes shuttle mutant SOD1, which was transported to the spinal neurons and attenuated neuronal cell death in ALS (Basso et al., 2013). As inflammation is one of the major factors that drive ALS, a study was designed to determine the interleukin-6 (IL-6) levels in astrocyte-derived exosomes of ALS patients. As a result, this study found a correlation between the rate of the disease progression and the IL-6 level, suggesting that astrocyte-derived exosomes in ALS patients could reveal the pathophysiology of the patients (Chen Y. et al., 2019). A study identified that neuronal-derived exosomes containing miR-124a could be up-taken by astrocytes and consequently promote glial glutamate Transporter 1 (GLT1) expression, which is involved in the pathological pathways of various CNS diseases such as ALS (Morel et al., 2013).

### Parkinson’s Disease

Level of α-synuclein is crucial in the pathogenesis of Parkinson’s disease (PD). Studies indicated that α-synuclein Transferring α-synuclein via astrocytes is of the ways that cells eradicate α-synuclein. Dysfunction in α-synuclein containing cells including astrocytes could be critical in the initiation or progression of PD and resolving these pathways may be a therapeutic innovation in PD (Stefanis et al., 2019). Previous studies suggested that uptake of exosomes containing α-synuclein by astrocytes can have disease-causing effects by the propagation of pathologic α-synuclein and changing astrocyte homeostasis (Sorrentino et al., 2019). Dopaminergic neurons are fragile to oxidative stress and astrocytes may protect neurons from iron-mediated toxicity which is caused by dopamine metabolic products in PD patients. Astrocytes were able to provide necessary biological molecules via exosomes to neurons in order to increase their resistance to oxidative damage. Inhibition of astrocyte-neuron communication could be the cause of PD (Ishii et al., 2019). For example, astrocyte-derived exosome miR-200a-3p prevents MPP^(+)^-induced apoptotic cell death through down-regulation of mitogen-activated protein kinase kinase 4 (MKK4) pathway (Shakespear et al., 2020). Besides, a recent study demonstrated the usefulness of neuron-derived, astrocytes-derived, and oligodendrocyte-derived exosomes in human plasma as a diagnostic biomarker for PD (Ohmichi et al., 2019).

### Cancer

Astrocyte-derived exosomes does not always have a positive effect on microenvironments and can be devastating. For example, astrocytes-derived exosomes enriched with miR-19a may increase brain cancer metastasis by increasing the loss of phosphatase and tensin homolog (PTEN) in tumor cells and C-C motif chemokine ligand 2 (CCL2) chemokine secretion (Zhang et al., 2015). Melanoma cells secrete EVs which can activate pro-inflammatory signals in astrocytes and promote metastasis and tumor formation (Gener Lahav et al., 2019). Glioma-derived EVs increase inhibition of TP53 and MYC signaling pathway activation in astrocytes. Those changes eventually result in pro-inflammatory development, tumor promotion, and progression (Hallal et al., 2019). Astrocytes develop tumor-like behaviors after being exposed to glioma EVs indicating that delivery of oncogenic material via EV may cause tumorigenic changes in astrocytes (Oushy et al., 2018). Previous research illustrated that drug-resistant cancers are caused by gene–gene, gene–miRNA, protein–protein signaling, and cell-to-cell interaction (Kitano, 2003, 2004). The transfer of genetic materials between cells in the tumor microenvironment, especially between tumor cells, has been demonstrated to promote tumor growth, invasion, and resistance to anti-tumor drugs (Mondal et al., 2017; Simon et al., 2018). A study found that glioblastoma patients’ blood samples showed that exosomal mRNA status could be related to treatment response (Shao et al., 2015). Other research found that miR-1238 was higher in drug-resistant glioblastoma cells and their exosomes than in drug-sensitive glioblastoma cells. This may indicate the important role of exosomal miR-1238 in mediating acquired drug resistance of glioblastoma cells (Yin et al., 2019). This is noteworthy because tumor-associated astrocyte-derived EVs contain *O6*-methylguanine-DNA methyltransferase (MGMT) mRNA which promotes anti-tumor resistance in glioma cells (Yu et al., 2018). Additionally, brain cancer cells communicate with astrocytes by exosomes. According to other research, it is also possible for glioblastoma cells to transfer oncogenic materials by exosomes to astrocytes in order to regulate the function of astrocytes (Rooj et al., 2016). Glioblastoma-derived exosomes could potentially convert normal astrocytes into a tumor-enhancing phenotype (Oushy et al., 2018). It was also reported that glioma cell-derived exosomes transfer long non-coding RNA, which was activated by transforming growth factor-beta (TGF-β) into astrocytes and cause miR-204-3p targeting in astrocytes and further promote the invasion of glioma cells (Bian et al., 2019). An *in vitro* study shown that EVs shed from U87 glioblastoma cell lines could degrade the gelatin matrix in astrocytes (Hallal et al., 2019).

As TGF-β is one of the glioblastoma biomarkers, achieving an early diagnosis of brain tumors may be possible by examining a patient’s body microfluid exosomes. Another study showed that a tumor suppressor, SAM, and SH3 domain-containing protein 1 (SASH1), was dysregulated or absent in glioma cells. Similar studies have also demonstrated the difference between the protein patterns of normal and glioma cells and the opposite effect of extracellular high-mobility group protein 1 (HMGB1) and astrocytic HMGB1 on SASH1 gene expression in one glioma cell line (Ma et al., 2019). Interaction between astrocyte-derived exosomes that contain neurotrophic induced cargoes such as co-chaperone stress-inducible protein 1 (STI1), and neuronal surface components could also enhance their pathological signaling (Hajj et al., 2013); STI1 also induced glioma through different pathways and is involved in neuronal death and neurodegenerative diseases (Erlich et al., 2007; Landemberger et al., 2018).

All these findings suggest that astrocytes may have the potential to become a therapeutic target in brain cancer patients.

### Other Diseases and Inflammation

Exosomes could also help to clarify the disease-causing factors and their pathophysiology in various CNS diseases. Astrocyte-derived exosomes could target neurons and contain neuroprotective proteins such as neuroglobin, which explained the neuroprotective role of astrocytes as well as the fact that they could exert their effect by secreting exosomes (Venturini et al., 2019). Besides, miRNAs in astrocytes-derived exosomes such as miR-26a could be potentially considered as a mediator for neuronal plasticity (Lafourcade et al., 2016). Studies have demonstrated that high levels of glial fibrillary acidic protein (GFAP) in MS patients during a clinical relapse and GFAP is expressed on astrocytes. It may be evidence that suggests the involvement of astrocytes in MS.

Exosome secretion of a psychosis-altered miRNA has been shown to regulate glutamate receptor expression. A research revealed that the inhibition of astrocytic miR-223 decreased the exosome mediated reduction in the expression of glutamate Receptor, Ionotropic, *N*-Methyl D-Aspartate 2B (Grin2b) in neurons. As a result, they found that this psychosis-altered miRNA can regulate the expression of glutamate receptors which are antagonized by antipsychotics (Amoah et al., 2019).

Nerve growth factor (NGF) protein is hyper-expressed in reactive astrocytes during spinal cord injury (SCI). The release of NGF could be mediated by exosomes and triggered by neuronal apoptosis post SCI. The appropriate balance of the nerve growth factor by reactive astrocytes may be a potential therapy for SCI (Cheng Y.Y. et al., 2019).

Traumatic brain injury (TBI) is also a common brain disease. As complement and proteins of astrocyte-derived exosome levels are considerably higher than those in neurons. These exosome could be considered as TBI biomarkers (Goetzl et al., 2020). Also, research indicates that astrocyte-derived exosome containing gap junction protein alpha 1- 20 kDa (GJA1-20k) could protect and repair the damaged neurons in TBI. Astrocytes-derived GJA1-20k protein could attenuate the connexin43 (CX43) phosphorylation, protect the mitochondria, decrease the cell death rate, and induce neuronal recovery (Chen W. et al., 2019).

It is noted that neurons and astrocytes could regulate each other through exosomes (Chaudhuri et al., 2018; Men et al., 2019; Venturini et al., 2019). Exosomes are transferred to neurons by glial cells including astrocytes and can cause neuroinflammation and depression through miRNA dysregulation (Brites and Fernandes, 2015).

Exosomes attenuated the inflammation by inhibiting the CXCL1 in astrocytes (Song et al., 2019). Interestingly, activated human astrocyte-derived EVs modulate neuronal uptake, differentiation, and firing (You et al., 2020). Astrocytes-derived EVs enriched IL-1β lower neurite development and maturation during neuroinflammation (You et al., 2020). Astrocytes may exert their inflammatory roles by utilizing exosomes (Sofroniew, 2015). Astrocytes could also induce inflammation on other cells through toll-like receptors (TLR) signaling and by exosomes enriched inflammation-inducing factors (Ibanez et al., 2019). Up-regulation of major histocompatibility complex1 (MHCI) molecules in astrocytes affect behavioral function by immune cascades, microglial proliferation, and neuronal numbers may lead to brain dysfunction in neuroinflammation-associated diseases, astrocytes may exert those inflammatory effects by releasing exosomes to target immune or neuronal cells (Sobue et al., 2018). IL-1β enriched astrocyte-derived EVs play an important role in cellular organization, cell communication, and inflammatory responses (You et al., 2020). Astrocyte-derived exosome containing HMGB1 can also be used as a biomarker in progression of inflammation in the brain of gulf war illness with drug treatment (Madhu et al., 2019).

## Conclusion and Future Perspectives

Despite of the recent efforts that have been made in understanding the role of astrocyte-derived EVs in CNS diseases, future studies are required to focus on the role of astrocyte-derived exosomes and their specific associated biological molecules as they are contained in both healthy and abnormal cells to resolve the functional effect of astrocyte secrete EVs. Identification of astrocyte-derived exosomes’ effects on both short and long-distance targets and their survival strategies may lead to the finding and development of new diagnostic and therapeutic methods. Further narrative researches on various biological molecules in astrocyte-derived exosomes or other brain cell derived-exosomes that modulate astrocytic function are required in order to further clarify their roles in the pathophysiology of various CNS diseases. Although cell-type-specific exosomes can be easily obtained *in vitro*, there is still difficulty *in vivo* study of EVs since a mix of different exosomes derived from different cells exist in extracellular space and it is not easy to distinguish them from one another. New approaches to label cell type specific exosomes are required which will be helpful for tracking cells in the study of intercellular communication *in vivo*. In this review, astrocyte-derived EVs have been demonstrated to carry or transfer biological molecules that are involved in both disease-causing and healing processes. Thus, astrocyte-derived EVs hold considerable potential for various clinical applications. Astrocyte-derived EVs are capable of crossing BBB and being up-taken by various brain cells, the ability of which makes them suitable to be used in drug targeted therapies a drug or drug carrier. The difference in the contents of astrocyte-secreted EVs make them suitable to be used as a biomarker for CNS disease diagnosis and progression of disease states. Study of astrocyte-derived exosomes leads to a better understanding of astrocytic functions in the brain. Despite the existence of plethora of evidence that supports the therapeutic potential of astrocyte-derived EVs, new *in vitro*/vivo models, more powerful imaging and tracking methods are required to track single astrocyte-derived EVs in the whole brain microenvironment in various disease and health situation in order to increase our understanding of astrocytes and their secreted EVs. Understanding the cell to cell communication via the study of astrocyte-derived exosomes and other brains cell-derived EVs may also contribute to brain development *in vitro*.

## Author Contributions

TG conceived, designed, and wrote the manuscript. G-YY and ZZ contributed to the discussion of ideas, helped in the correction, and proofread the manuscript. All the authors contributed to the article and approved the submitted version.

## Conflict of Interest

The authors declare that the research was conducted in the absence of any commercial or financial relationships that could be construed as a potential conflict of interest.

## Abbreviations

AD: Alzheimer’s disease
ALS: amyotrophic lateral sclerosis
AMPK/mTOR: AMP-activated protein kinase and Rapamycin
BBB: blood-brain barrier
BM: bone marrow
CCL2: C-C motif chemokine ligand 2
CNS: central nervous system
CTGF: connective tissue growth factor
CXCL1: the chemokine (C-X-C motif) ligand 1
CX43: connexin43
EV: extracellular vesicle
ECM: extracellular matrix
GFAP: glial fibrillary acidic protein
GJA1-20k: gap junction protein alpha 1- 20 kDa
GLT1: glial glutamate transporter 1
Grin2b: glutamate receptor, ionotropic, *N*-methyl D-aspartate 2B
Hsp/c70: heat shock protein/C-terminal70
HMGB1: high-mobility group protein 1
IL-1β: interleukin 1beta
IL-10: interleukin 10
IL-6: interleukin 6
MGMT: *O*6-methylguanine-DNA methyltransferase
MHC1: major histocompatibility complex1
miRNA: microRNA
MKK4: mitogen-activated protein kinase kinase 4
MSC: mesenchymal stem cell
MS: multiple sclerosis
MV: micro-vesicles
NGF: neuron growth factor
Nrf2-NF- κ B: nuclear factor (erythroid-derived 2)-like 2 and nuclear factor-κ B
NSC: neuronal stem cell
OGD: oxygen glucose deprivation
PD: Parkinson’s disease
PTEN: phosphatase and tensin homolog
TBI: traumatic brain injury
TGF-β: transforming growth factor beta
TLR: toll-like receptors
RABEPK: Rab9 effector protein with Kelch Motifs
SASH1: SAM and SH3 domain-containing protein 1
SCI: spinal cord injury
SOD1: superoxide dismutase 1
STI1: stress-inducible protein 1.
